# Multisensory mental representation of objects in typical and Gifted Word Learner dogs

**DOI:** 10.1007/s10071-022-01639-z

**Published:** 2022-06-08

**Authors:** Shany Dror, Andrea Sommese, Ádám Miklósi, Andrea Temesi, Claudia Fugazza

**Affiliations:** 1 Department of Ethology, Eötvös Loránd University, Pázmány P. s 1c, 6th Floor, 1117 Budapest, Hungary; 2 Doctoral School of Biology, Institute of Biology, ELTE Eötvös Loránd University, Budapest, Hungary; 3 MTA-ELTE Comparative Ethology Research Group, Budapest, Hungary

**Keywords:** Object discrimination, Object mental representation, Object recognition, Olfaction, Sensory modalities, Vision

## Abstract

Little research has been conducted on dogs’ (*Canis familiaris*) ability to integrate information obtained through different sensory modalities during object discrimination and recognition tasks. Such a process would indicate the formation of multisensory mental representations. In Experiment 1, we tested the ability of 3 Gifted Word Learner (GWL) dogs that can rapidly learn the verbal labels of toys, and 10 Typical (T) dogs to discriminate an object recently associated with a reward, from distractor objects, under light and dark conditions. While the success rate did not differ between the two groups and conditions, a detailed behavioral analysis showed that all dogs searched for longer and sniffed more in the dark. This suggests that, when possible, dogs relied mostly on vision, and switched to using only other sensory modalities, including olfaction, when searching in the dark. In Experiment 2, we investigated whether, for the GWL dogs (N = 4), hearing the object verbal labels activates a memory of a multisensory mental representation. We did so by testing their ability to recognize objects based on their names under dark and light conditions. Their success rate did not differ between the two conditions, whereas the dogs’ search behavior did, indicating a flexible use of different sensory modalities. Little is known about the cognitive mechanisms involved in the ability of GWL dogs to recognize labeled objects. These findings supply the first evidence that for GWL dogs, verbal labels evoke a multisensory mental representation of the objects.

**Supplementary Information:**

The online version contains supplementary material available at 10.1007/s10071-022-01639-z.

## Introduction

Search tasks, in which one is requested to find a specific stimulus, may rely on discrimination or recognition. We refer to discrimination when an individual perceives the difference between two (or more) stimuli/objects and expects them to result in different outcomes (Blair et al. 2003). Recognition occurs when the subject identifies a stimulus as one that s/he has previously encountered (Akkerman et al. 2012). Recent studies have confirmed that dogs can discriminate, for example, among visual images (Range et al. 2008), images of dogs from other animal species (Autier-Dérian et al. 2013), human voices (Gábor et al. 2019), and olfactory stimuli (Pinc et al. 2011).

In human infants, visual object discrimination develops earlier than object recognition and it is hypothesized that these two processes involve different neural circuits (Overman et al. 1992). The performance of both human infants (Overman et al. 1992) and dogs (Milgram et al. 1994) in object discrimination and recognition tests suggests that the latter is a more complex task. Moreover, when solving object recognition tasks, dogs require a large number of trials to achieve predetermined learning criteria (Milgram et al. 1994).

According to cognitive computational theories, perceptual information is processed in the mind to form mental representations of the environment (Sternberg 2009). In humans, the information obtained from different perceptual modalities is integrated, leading to the formation of a multisensory mental representation (Lacey et al. 2007). In dogs, studies have shown similar modalities used to develop a multisensory representation of social stimuli. Adachi et al. (2007) argued that dogs form a multisensory representation of their owners. They found that, when tested in a violation of the expectations paradigm, dogs looked longer when the presented face did not match the audio recording that was played. In another study, dogs were presented with women and men while listening to a recording of a human voice. Dogs that lived with both genders looked longer at the person whose gender matched the played recording (Ratcliffe et al. 2014).

Studies on the sensory modalities used by dogs during search tasks reported that dogs showed a tendency to rely on visual information (Bräuer and Belger 2018) or a combination of vision and olfaction to find their target (Polgár et al. 2015). Kaminski et al. (2009) found that while engaging in an object recognition task, some dogs were able to rely purely on visual information, as they identified objects from pictures. Explosive detection dogs were able to find their target under complete darkness, demonstrating that they could discriminate between stimuli by relying only on olfactory cues (Gazit and Terkel 2003). In addition, there is evidence that dogs can use tactile information to categorize objects (van der Zee et al. 2012). However, overall, only a few studies investigate the abilities of dogs to use sensory modalities other than vision and olfaction (Bálint et al. 2020).

Few dogs present the rare ability to identify objects based on their verbal labels (Kaminski et al. 2004; Pilley and Reid 2011; Fugazza et al. 2021a, b). We labeled these dogs as Gifted Word Learner (GWL) dogs (Fugazza et al. 2021b). The identification of objects based on their verbal labels can be considered a specific case of object recognition. Just like humans, GWL dogs not only recognize the labeled objects—or categories of objects (Fugazza and Miklosi 2020) as stimuli they have already encountered, but they also identify them among other similarly familiar named objects, based on their verbal labels. It is unknown whether the extreme difference between typical dogs (hereafter, T dogs) that lack this capacity, and GWL dogs rises from differences in the ability to discriminate and/or recognize objects, or whether it derives from constraints related to associating labels to objects (Ramos and Mills 2019).

Language acquisition is not fundamental for forming a cross-modal mental representation of objects, however, familiarization with objects' verbal labels might facilitate the process (Lacey et al. 2007). Therefore, in Experiment 1, we investigated the capacity and sensory modalities used by T and GWL dogs to discriminate objects recently associated with a reward from distractors, under light and dark conditions. Previous studies have shown that dogs form multisensory mental representations of social stimuli and that, in the absence of specific training, they tend to rely on vision or vision and olfaction during search tasks. We, therefore, hypothesized that depending on the environmental constraints, the dogs will rely on different sensory modalities, and will successfully discriminate the objects used in this test. More specifically, we hypothesized that under the conditions tested in this experiment dogs will mostly rely on vision, when possible, but they will successfully switch to using other sensory modalities in the dark. Thus, we predicted that their searching behavior, but not their overall success rate, will differ between light and dark conditions. Based on the evidence of typical dogs’ discrimination capacities (Affenzeller et al. 2017; Milgram et al. 2005), we expected that both GWL and T dogs would solve the discrimination task. However, as it is not clear to which extent the verbal labels of the objects influence their mental representations, the two groups may differ in their searching behavior.

In Experiment 2, we utilized the GWL dogs' pre-existing vocabulary of object names to examine whether the object verbal label elicits the recall of a multisensory mental representation. We hypothesized that upon hearing the verbal label of an object, the GWL dogs recall a specific multisensory mental representation so that their recognition capacity is not affected by the lack of visual information. Hence, we predicted that, when searching for a named object, their success rate does not differ between light and dark conditions, while the sensory modalities used to recognize it do.

## Materials and methods

### Experiment 1

#### Subjects

We tested 14 dogs, 10 of which were typical (T) family dogs (5 males, 5 females, age = 2.8 years ± 1.8) and 3 were GWL dogs (1 male, 2 females, age = 2.9 years ± 2.8). The T dogs were from various breeds (5 Border Collies, 1 Pinscher, 1 Labrador-poodle cross, 1 mongrel, 1 Australian Shepherd, and 1 Border Terrier). They were selected based on their owners' reports that they were motivated to retrieve toys but did not have knowledge of object names or experience in scent detection. The GWL dogs participating were all Border Collies. These dogs (Max, Gaia, and Nalani) had participated in a previous study (Fugazza et al. 2021b) and proved to know the names of more than 20 dog toys (for the methods and results see Fugazza et al. 2021b).

## Procedure

*Location* 1 GWL dog and 10 T dogs were tested at the Department of Ethology at ELTE University, Budapest, Hungary. The dogs were familiar with this location as they had participated in previous, unrelated experiments. 3 of the GWL dogs were tested at their homes (Whisky in Norway, Nalani in Nederland, Gaia in Brazil) using an experimental setup that was like the one available in the lab (see *setup* section below).

**Fig. 1:**
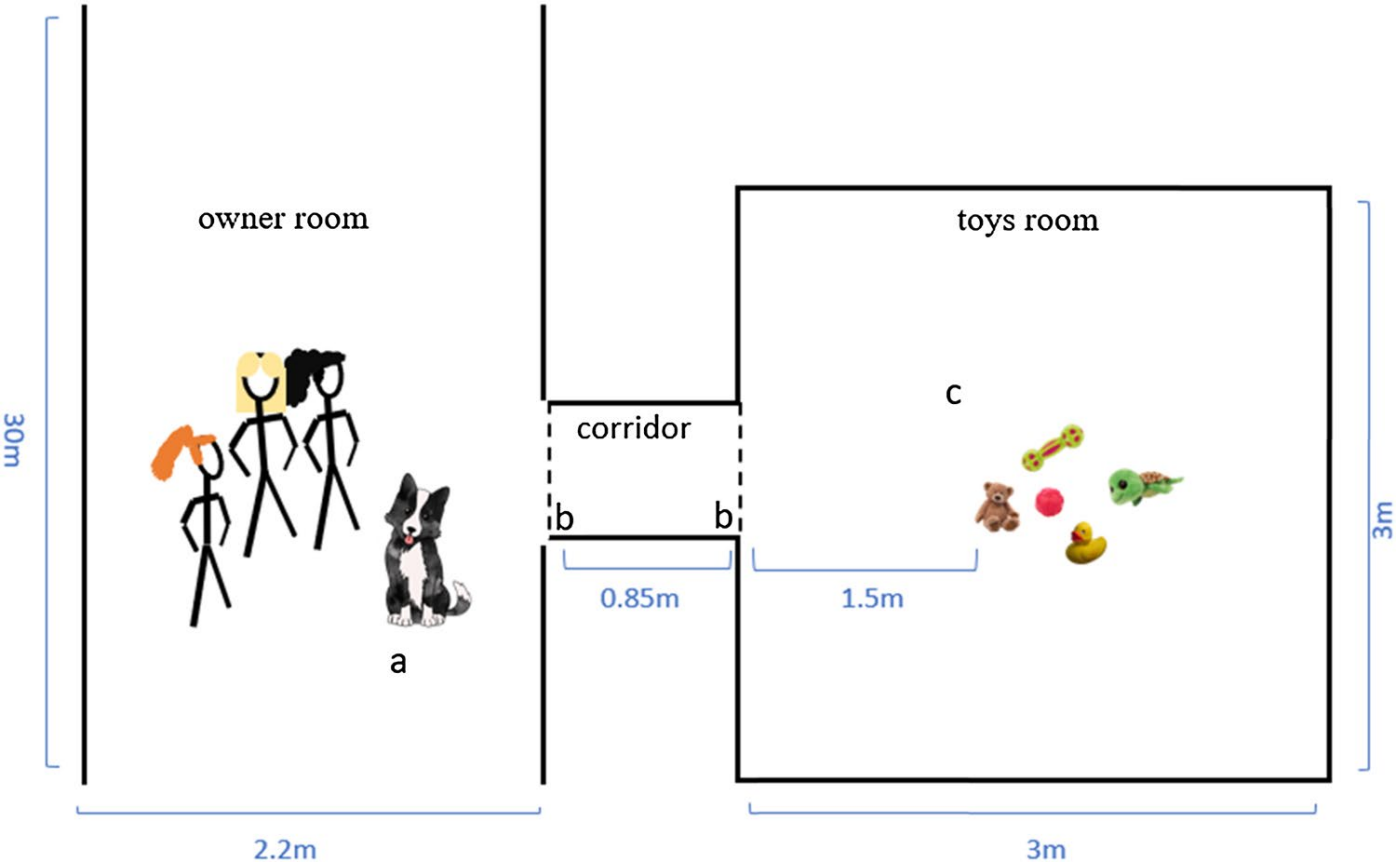
The experimental setup. **a** The dog, its owner, and experimenters were positioned in the owner's room; **b** Two sets of heavy curtains (dotted lines) were hung at both openings of the corridor to prevent light from the owner's room from entering the toys room; **c** The toys were positioned out of the owners view in the toys room. Measurements in the figure are from the laboratory of ELTE University. For the two GWL dogs that were tested at their owners' homes, the experimental setup was identical, but the room measurements were different (see appendices)

*Setup* The experimenter (E) and the dog owner (O) stood with the dog in one room (owner room) while the toys were placed in an adjacent room (toys room). A corridor connected the two rooms and heavy curtains were hung in both openings of the corridor. These curtains prevented light from the owner's room from entering the toy room. All the windows in the toy room were covered with multiple layers of dark nylon sheets to prevent external light from entering the room (Fig. 1).

*Objects* For all dogs, the same 10 unfamiliar objects (dog toys) were used during the experiment. The toys were of different shapes, sizes, materials, and colors (see Fig. 1 in the supplementary material). For each dog, E randomly divided the 10 toys into two sets and randomly selected a toy out of each set to serve as a target toy (target toys 1 and 2). The additional four toys in each set served as distractor objects. The allocation of the toys to serve as a target or a distractor was random across dogs (a toy that served as a target toy for one dog served as a distractor for a different dog).

*Training* E gave the target toy to the owner (target toy 1). O then played with it with the dog, occasionally placing it among the 4 other distractor toys, and rewarding the dog with praise, play, and/or food, when it retrieved it. The training duration was between 5 and 10 min. For a detailed description of the training procedure, see appendices.

After the training, the dog received a 5-min break and continued to the light baseline test to assess the training success (see below). The same target toy was also used in the dark condition (see Dark condition below). After the dog completed both conditions, on a separate testing occasion, the whole process was repeated using a different target toy (toy 2). 1 day to two weeks elapsed between the two testing occasions, depending on the owners’ availability. For each subject, the toys were randomly assigned to serve as toys 1, 2, or distractors. Overall, each dog was tested twice in the light baseline (once with toy 1 and once with toy 2) and twice in the dark condition (once with toy 1 and once with toy 2). For pictures of the toys see Fig. 1 in the supplementary material.

## Light condition

*Testing procedure* The dogs were requested to retrieve the target toy when it was placed among 4 other toys used as distractors during the training stage. The toys were randomly scattered on the floor in an area of about 1.5 m in diameter. In each trial, O asked the dog to fetch the target toy (e.g., “Go get it!”). The test consisted of 10 trials. After every successful trial, the dog was rewarded by playing with the retrieved toy, praise, and/ or food, then E took the toy back to the toys’ room and shuffled all the toys on the floor. If the dog made an incorrect choice, O did not reward the dog and gave the retrieved toy back to E, who repeated the procedure described above. If the dog failed to retrieve the correct toy in 7/10 trials, it repeated the pretraining stage with a different target toy.

## Dark condition

*Setup and testing procedure* The test setup and procedure were identical to the light baseline but the lights in the corridor and the toys’ room were turned off. When the dog passed from one room to the other, the curtains hanging at the entrances of the room prevented the transmission of light. Light measurements conducted with Luxmeter (VOLTCRAFT MS-1300®) confirmed that there was complete darkness (lux = 0) in the toys’ room.

## Data collection

The tests were recorded using an infrared video camera (Sony® Exmor R Balanced Optical Steady Shot 30X). The footage was coded using Solomon Coder beta 19.08.02 (Copyright © 2010 András Péter; http://solomoncoder.com, Eötvös Loránd University, Budapest, Hungary). The dogs' correct or incorrect object choices were marked for all trials. In addition, the behavior of the dogs in the toys room was coded. As behavioral coding was time-consuming, for each dog, we coded the first and the last three trials of each condition, using the following behavioral variables (see also Supplementary video).

*Object choice* We considered a toy to be chosen by the dog when it exited the toy room with it in its mouth. We coded this as a binary variable: 1 = the dog selected the correct object; 0 = the dog did not select the correct object.

*Search* The dog oriented its head towards the floor, carrying the head in line with the shoulder blades or lower. If the dog picked up a toy, lifted the head higher than the shoulder blades, or stopped orienting towards the floor and the toys, the measurement of this behavior was interrupted until the dog resumed the searching position described above. We measured the duration of this behavior.

*Sniffing* The dog’s sniffing behavior was coded every time that the sound of the dog inhaling through the nostrils was heard by the coders. This behavior was coded only when the dog was also engaged in search behavior. For this behavior, we measured the frequency and duration.

*Straight approach* The dog entered the toy room and moved towards a toy in a straight line, without diverting the head to the sides, until picking up the toy. We measured the frequency of straight approaches.

*Picking up an object* The dog picked up an object with its mouth. We measured latency to pick the object up from the moment the dog entered the toy room. Picking an object up also marked the end of the searching behavior, unless the dog dropped the toy and kept searching.

*Mouthing* The dog chewed a toy or shook it. We measured the duration of mouthing. This variable was included as an indication of the use of tactile and gustatory senses.

Twenty percent of the data was coded by an independent coder to determine inter-rater agreement.

## Data analysis

For the behavioral analysis, we coded the *object choice* and *straight approach* as separate binary responses (i.e., 1 = correct choice or straight approach, 0 = incorrect choice or not straight approach). The durations and latencies were measured in seconds. Statistical analyses were carried out in the R environment (R Core Team 2019). The latency to *picking up an object* was analyzed in Cox Mixed Models (CMM). The probability of correct choice (binary response) in Experiment 1 was analyzed using a binomial test, with the chance level set at 0.2 as there were always 5 toys to choose from. The subsequent analyses of all other behavioral responses described above included the first 3 and last 3 trials. Cronbach’s alpha was used to assess the inter-observer reliability of the two independent coders (DeVellis 1991). Behavioral responses were analyzed in separate Linear Mixed Models (LMM, for durations and frequencies, Pinheiro et al. 2019) and binomial Generalized Linear Mixed Models (GLMM, for binary responses; Bates et al. 2014). Initial models included ‘trial’ (factor with 6 levels: 1–3 and 8–10) and ‘dog group’ (factor with two levels: T and GWL dogs). Since there was no difference between the first and last trials and the two dog groups did not differ in any of the response variables (see “Results”), both explanatory variables were excluded from the final models. GLMMs included condition (Light or Dark) and toy (1 and 2) as explanatory variables. Finally, the dogs’ names were used as random effect in the model.

## Results

### Inter-rater agreement was excellent for all the variables (Cronbach’s alpha, all variables > 0.9)

**Fig. 2:**
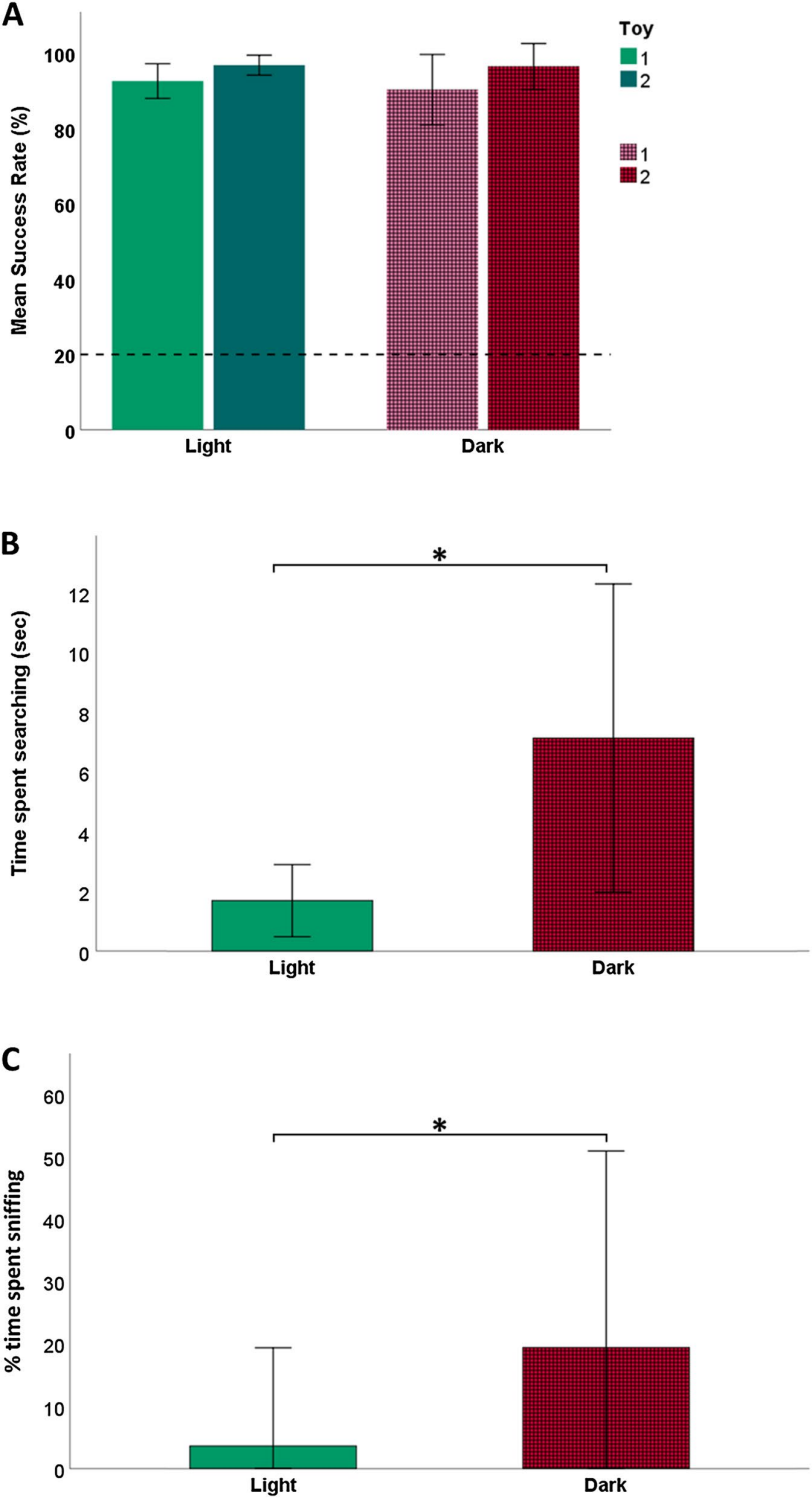
**a** Mean success rate (± SD) of all dogs (irrespective of type, i.e., GWL or T) in choosing the correct toy in light and dark conditions. Separate bars illustrate success toys 1 and 2. The dashed line represents the chance level at 20%. **b** The mean time spent searching (± SD, in seconds) in the two conditions. **c** Percentage of searching time spent sniffing (± SD) in the light and dark conditions. Significance (GLMM, *p* < 0.05) is indicated with *

All dogs, except for one T dog (Scotch), reached the a priori set criterion in the light baseline test (7/10 correct trials) after the first attempt. Scotch succeeded after repeating the training and the test with a new object (binomial test,* p* < 0.05, Table S1 in the Supplementary material). All dogs were individually successful well above chance level (binomial tests, all p < 0.05, Table S1 in the supp. mat.) in both light baseline and dark conditions, with both toys 1 and 2 (Fig. 2a).

The dogs’ success rate was always above chance (*z* = 7.899, *p* < 0.001) and there was no difference between the two groups (*χ*2 = 0.701, *df* = 1, *p*  = 0.791). GWL and T dogs did not differ significantly in their behavioral response between the beginning (first 3 trials) and the end (last 3 trials) of the test (*χ*2 = 4.616, *df* = 5, *p*  = 0.465). In addition, the two groups did not differ in any of the other response variables (LRT of dog group, LMM of frequency of sniffing: *χ*2 = 0.051, *df *= 1, *p* = 0.820; GLMM of frequency of straight approach: *χ*2 = 0.074, *df* = 1, *p* = 0.785; CMM of latency to pick up the toy: *χ*2 = 1.33, *df* = 1, *p* = 0.249; LMM of duration of sniffing *χ*2 = 0.923, *df* = 1, *p* = 0.337, searching *χ*2 = 0.359, *df* = 1, *p* = 0.549; and mouthing *χ*2 = 0.262, *df* = 1, *p* = 0.608), hence we analyzed results of all dogs together, irrespective of dog type. Accuracy in choosing the target toy was not influenced by the condition (*χ*2 = 0.239, *p* = 0.625). There was an order effect related to the success rate: dogs showed a higher success rate with Toy 2 – i.e., the toy used in the second instance (*χ*2 = 5.473, *df* = 1, *p* = 0.01). Although, there was never a significant difference between toy 1 and toy 2 in relation with the other behavioral variables (all *p*-values > 0.05). Thus, we also discarded this variable from the model.

There was a significant difference between conditions, with dogs spending more time searching (*χ*2 = 122.92, *df* = 1, *p* < 0.001; Fig. 2b) and longer latency to pick up the toy in the dark (*χ*2 = 53.393, *df* = 1, *p* < 0.001). The duration of mouthing did not differ between conditions (*χ*2 = 1.653, *df* = 1, *p* = 0.197). The condition also affected the frequency of straight approach, which never occurred in the dark (*χ*2 = 75.394, *df* = 1, *p* < 0.001).

The proportion of searching time spent sniffing was different between conditions, with the dogs spending more time sniffing while searching in the dark (*χ*2 = 18.989, *df* = 1, *p* < 0.001; Fig. 2c).

## Experiment 2

### Subjects

The 3 GWL dogs tested in experiment 1 were also tested in this experiment, as well as an additional female Border Collie (Whisky, 4.4 years old).

## Procedure

*Location and setup* The location and setup were as described for experiment 1.

*Objects* Each of the GWL dogs possessed a collection of familiar and named dog toys. The 4 dogs’ knowledge of these object names was confirmed in Fugazza et al. (2021b). For each dog, 20 of these toys were randomly chosen and scattered on the floor in a surface area of about 3 m in diameter.

## Light condition

*Procedure* E instructed O to ask the dog to retrieve a toy by pronouncing the toy’s name. The dog then left the owner's room and entered the toy room to select a toy. If the dog successfully retrieved the correct toy, it was rewarded with play, praise, and food. If the dog made a mistake, the trial was repeated but the results of the repeated trials were not included in the analysis of the success rate. If the dog made another consecutive mistake, E instructed O to proceed with the next trial. The order of the toys was randomly determined. After every five trials, E placed 5 additional randomly selected toys on the floor. This way, the number of toys from which the dog could choose always varied between 20 and 16.

## Dark condition

The test was identical to the light baseline test, but the lights in the toys’ room and corridor were turned off.

## Data collection

The dog's correct or incorrect choice was coded in all trials. The behavioral variables described for experiment 1 were also coded in experiment 2 for all trials.

## Data analysis

Statistical analysis was carried out similarly to that of Experiment 1, except that, for the analysis of the success rate, the chance level was conservatively set at 0.06 because the total number of toys available ranged between 16 and 20.

## Results

Inter-rater agreement was again excellent for all the variables (Cronbach’s alpha, all variables > 0.9).

**Fig. 3:**
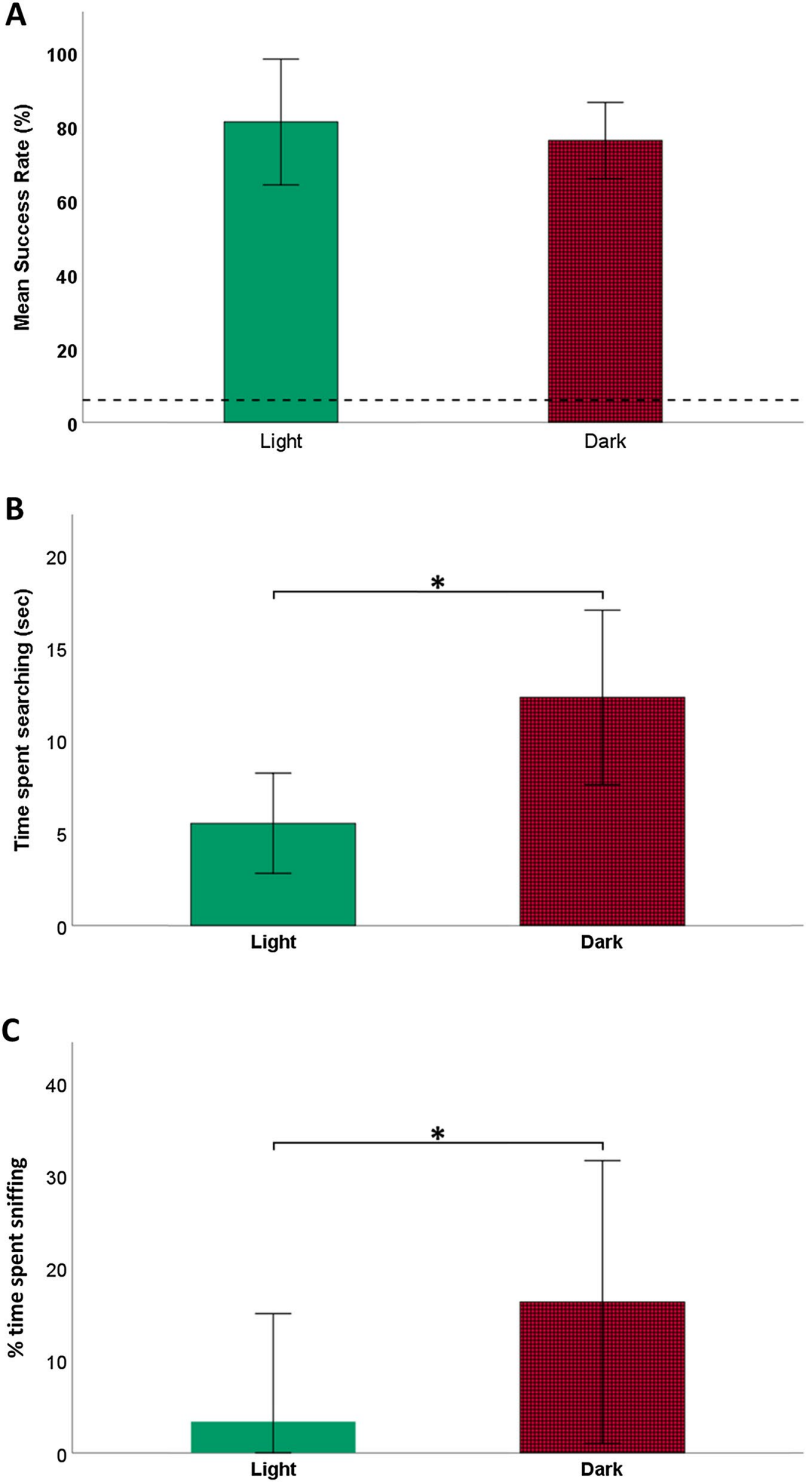
**a** The bars show the mean success rate (± SD) of the GWL dogs in both conditions. The dashed line represents the chance level (6%, determined based on the lowest number of toys present for the dogs to select from). **b** The mean time spent searching (± SD, in seconds) in the two conditions. **c** Percentage of searching time (± SD) spent sniffing in the light and dark conditions. Significance (GLMM, *p* = 0.05) is indicated with *

The GWL dogs successfully selected the correct toy in both light and dark conditions (binomial test, all *p* < 0.05, Table S2 in the supplementary material), with no significant difference between the two (GLMM: *χ*2 = 2.049, *df *= 1, *p* = 0.152; Fig. 3a).

The GWL dogs spent more time searching for the named toys in the dark condition compared to the light baseline (*χ*2 = 9.255, *p* < 0.001; Fig. 3b); There was no significant difference between conditions for the latency to pick up the toy (*χ*2 = 0.152, *p* = 0.696), and duration of mouthing (*χ*2 = 0.046, *p* = 0.831).

We did not observe any straight approach in the dark condition, while we observed straight approaches in 15 trials out of 80 (1 for Gaia, 2 for Max, 4 for Nalani, and 8 for Whisky) in the light baseline.

The proportion of searching time spent sniffing was different between conditions with the dogs spending more time sniffing while searching in the dark (*χ*2 = 3.671, *df* = 1, *p* < 0.05; Fig. 3c).

## General discussion

While the dogs' success in both experiments did not differ between conditions, our detailed behavioral analysis revealed that, when searching in the dark, dogs spent a longer time actively searching and sniffed more.

These findings indicate that dogs integrated information perceived through different sensory modalities and that, while vision was among the preferred modality for identifying the objects tested in this experiment, dogs can spontaneously and successfully revert to using only other senses if visual information is not available. By doing so, dogs present a flexible use of different sensory modalities (see also Szetei et al. 2003; Polgár et al. 2015).

The occasional straight approaches observed only in the light baseline suggest that, when visual information is available, dogs can also identify the object from a distance. However, most often, dogs tended to search among the different objects from a closer distance. This indicates the use of close-range vision and also, potentially, other sensory modalities, including not only olfaction but also touch—as we found very few and short occurrences of sniffing in the light baseline. Our results are consistent with the findings of Bräuer and Belger (2018), who observed that sniffing behavior increased the latency of approaching and decreased the number of direct approaches towards a target object.

Humans can rely on tactile information when visual input is limited (Lacey et al. 2007). Nevertheless, our results did not reveal differences in the time spent by the dogs exploring the toys with their mouth (i.e., mouthing behavior) between conditions in both experiments. This may indicate that these senses are equally used, irrespectively of the illumination, or that they are not relied on at all in object search. However, dogs may also display behaviors other than what we defined as “mouthing” when using tactile or gustatory senses, such as using their noses or whiskers. Thus, we do not exclude that these sensory modalities may have been used differently in the two experimental conditions by the dogs. In addition, dogs often mouth toys as part of their play behavior. It could, therefore, be that the definition of this behavioral variable was not sensitive enough to reflect the use of tactile sensation.

In Experiment 1, all dogs displayed a high success rate, that did not differ between conditions. This demonstrates that both T and GWL dogs can discriminate between a target object, associated with a reward during the immediately preceding training, and distractor objects. These findings are in agreement with previous studies reporting on dogs’ ability to perform object discrimination tasks (Milgram et al. 1994; Head et al. 1998; Tapp et al. 2004) and expand those to situations of limited sensory information. Our finding that although the dogs’ success rate in Experiment 1 was already above chance when tested on the first toy (i.e., toy 1), their performance increased when tested again (i.e., on toy 2), could be attributed to the dogs becoming experienced in the task and familiar with the test situation during the experiments (Hunter and Kamil 1971). Similarly, Bräuer and Belger (2018), described that dogs’ latency of finding a target object decreased as their experience in the task increased.

We did not find differences between the success rate of T and GWL dogs in the object discrimination task, nor did we observe differences in their searching behavior. This suggests that the extreme difference between the ability of GWL and T dogs to recognize objects based on their labels (Fugazza et al. 2021a, b) does not result from differences in object discrimination capacities.

While in Experiment 1, the two groups of dogs discriminated rewarded from non-rewarded objects, in Experiment 2, the objects from which the GWL dogs had to select were all familiar objects. Thus, this is a specific complex case of object recognition that cannot be solved by simply relying on familiarity. The GWL dogs' success in recognizing these objects according to their verbal labels did not differ between dark and light conditions. Ganea (2005) described how, after hearing the names of familiar objects, 14-month-old infants started to search for them and found the objects, thereby demonstrating that the objects’ verbal labels led to the retrieval of the object’s representation. When tested in the object recognition task, GWL dogs demonstrated that they can recognize familiar objects under limited sensory inputs, thereby demonstrating that they have formed a multisensory mental representation of the object (Lacey and Sathian 2011, for review). Moreover, the GWL dogs’ success in retrieving the named toys shows that for each object verbal label, they form a specific multisensory mental representation, enabling them to recognize the correct toy even when it is placed among other labeled objects in the dark. In other words, for GWL dogs, hearing an object’s verbal label evokes a mental representation of the object.

To summarize, we found that, in the absence of formal training, dogs mostly rely on proximate vision and, potentially, touch sense in object discrimination and recognition tasks but can switch to using only other sensory modalities when vision is not possible. Dogs spontaneously encode different features of the objects, leading to the construction of multisensory mental representations. In the case of GWL dogs, a memory of the multisensory representation is evoked by hearing the objects' verbal labels as they perform complex object recognition tasks.

## Supplementary Information

Below is the link to the electronic supplementary material.

Supplementary file1 (DOCX 237 kb)

Supplementary file2 (MP4 9477 kb)
