# Supplementary material for: Multisensory mental representation of objects in typical and Gifted Word Learner dogs
Source: Anim Cogn. 2022 Jun 8;25(6):1557–66. doi: 10.1007/s10071-022-01639-z (PMC9652232; doi:10.1007/s10071-022-01639-z)
Supplement: Supplementary file 1 — Supplementary file1 (DOCX 237 kb) [file 10071_2022_1639_MOESM1_ESM.docx]

# Supplementary information:

Training procedure:

E randomly selected 1 toy out of a group of 5, to serve as a target object and gave it to O, who presented it to the dog. O played with the dog with the toy by throwing it and rewarding the dog with praise, play, and/ or food for retrieving it. The training then proceeded in the following difficulty levels:

1. E placed the remaining 4 toys on the floor and O threw the target toy so it landed between the 4 other distractor objects. O then asked the dog to retrieve the toy and rewarded it for a correct choice.
2. O stepped out of the room, into the corridor, and threw the toy again into the room, so it landed among the 4 distractor objects. O then asked the dog to retrieve the toy and rewarded it for a correct choice.
3. While standing in the corridor with the dog, O showed the dog that it was giving the toy to E, who went into the room and placed it among the 4 distractor objects. O then asked the dog to retrieve the toy and rewarded it for a correct choice.

If the dog successfully retrieved the toy, it proceeded to the subsequent training level. If the dog made a mistake, O entered the room, pointed at the target toy, and encouraged the dog to pick it up. The entire training procedure took between 5 to 10 minutes, after which the dog received a 5-minute break before continuing to the light baseline test.

A video demonstrating the behavioral variables coded is available at:

<https://youtu.be/VmqH-xCzhFA>


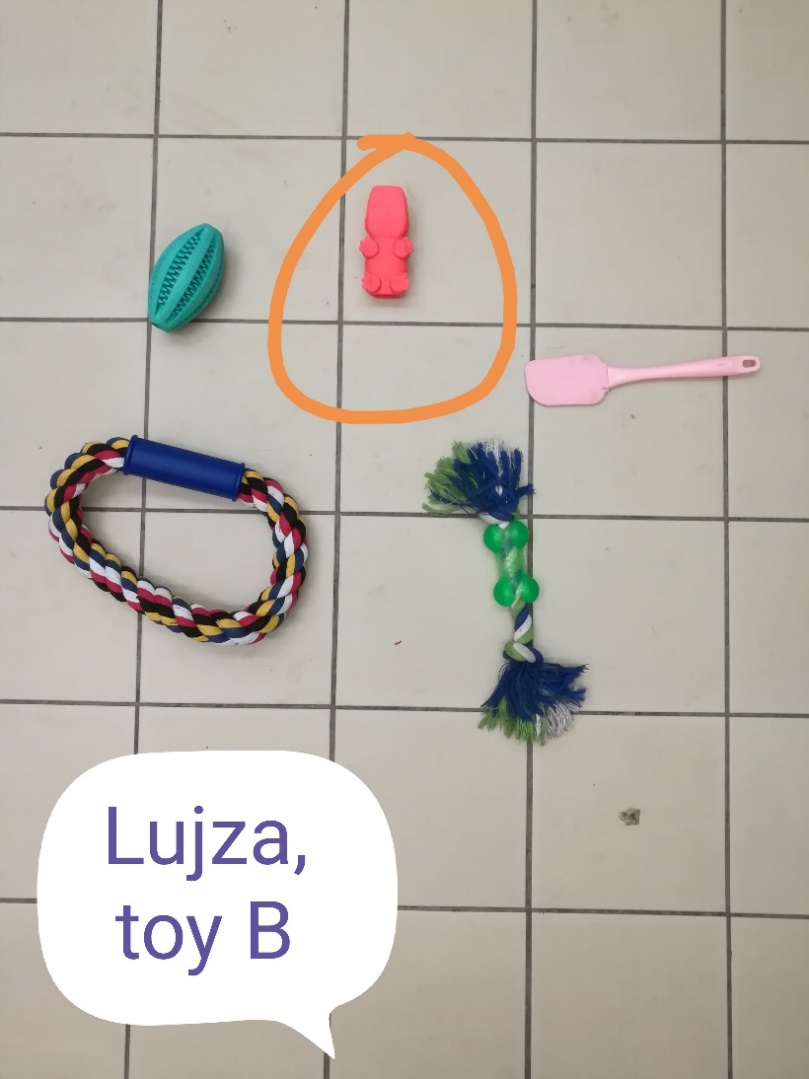

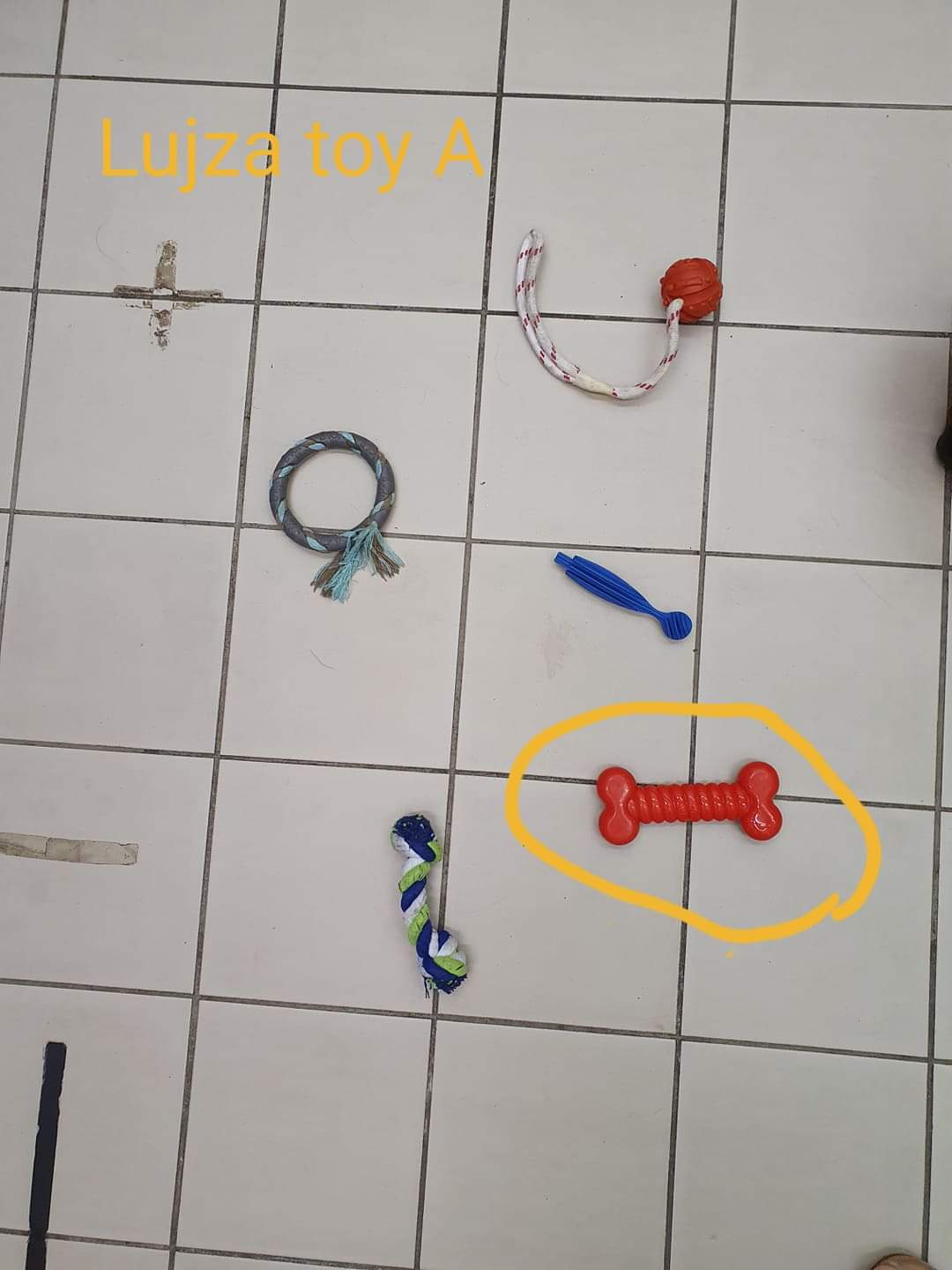


Figure 1: Pictures of the disposition of the toys on the floor for Experient 1. Randomly chosen target toys are marked with a circle and are randomly placed among 4 other distractor toys.

| **ID** | **Light A** | **p-value** | **Dark A** | **p-value** | **Light B** | | **p-value** | | **Dark B** | **p-value** |
| --- | --- | --- | --- | --- | --- | --- | --- | --- | --- | --- |
| Bjorn | 80% | < 0.001 | 60% | 0.006 | | 90% | | < 0.001 | 60% | 0.006 |
| Borisz | 100% | < 0.001 | 100% | < 0.001 | | 100% | | < 0.001 | 100% | < 0.001 |
| Frizbi | 90% | < 0.001 | 100% | < 0.001 | | 90% | | < 0.001 | 100% | < 0.001 |
| Kesu | 100% | < 0.001 | 100% | < 0.001 | | 100% | | < 0.001 | 100% | < 0.001 |
| Kiki | 90% | < 0.001 | 100% | < 0.001 | | 100% | | < 0.001 | 100% | < 0.001 |
| Lizy | 80% | < 0.001 | 50% | < 0.001 | | 100% | | < 0.001 | 50% | 0.033 |
| Lujza | 100% | < 0.001 | 80% | < 0.001 | | 100% | | < 0.001 | 100% | < 0.001 |
| Mokka | 100% | < 0.001 | 100% | < 0.001 | | 90% | | < 0.001 | 100% | < 0.001 |
| Scotch | 80% | < 0.001 | 90% | < 0.001 | | 100% | | < 0.001 | 100% | < 0.001 |
| Zeno | 100% | < 0.001 | 100% | < 0.001 | | 100% | | < 0.001 | 100% | < 0.001 |
| Gaia | 100% | < 0.001 | 100% | < 0.001 | | 100% | | < 0.001 | 100% | < 0.001 |
| Max | 90% | < 0.001 | 95% | < 0.001 | | 100% | | < 0.001 | 100% | < 0.001 |
| Nalani | 100% | < 0.001 | 100% | < 0.001 | | 90% | | < 0.001 | 100% | < 0.001 |

Table 1: Percentage of correct trials and p-values (binomial test) for each dog in light and dark testing conditions for Toy A (Light A and Dark A) and Toy B (Light B and Dark B). In all the tests, the chance level was always set at 0.2 as 5 toys were always present to choose from.

| **ID** | **Light** | **p-value** | **Dark** | **p-value** |
| --- | --- | --- | --- | --- |
| Gaia | 60% | < 0.001 | 65% | < 0.001 |
| Max | 95% | < 0.001 | 75% | < 0.001 |
| Nalani | 75% | < 0.001 | 75% | < 0.001 |
| Whisky | 95% | < 0.001 | 90% | < 0.001 |

Table 2: Percentage of correct trials and p-values (binomial test) for each GWL dog in the light and dark testing conditions of Exp. 2.
